# The developmental changes of fecal microbial composition and diversity in emu (*Dromaius novaehallandiae*) at early growth stages

**DOI:** 10.3389/fmicb.2025.1744168

**Published:** 2026-01-12

**Authors:** Leru Deng, Xinyi Chen, Shuhan Pan, Wenling Huang, Yucheng Yin, Xueyan Wei, Huihua Zhang, Cui Zhu

**Affiliations:** School of Animal Science and Technology, Foshan University, Foshan, China

**Keywords:** 16S rRNA gene sequencing, EMU, fecal microbiota, microbial diversity, ontogenetic development

## Abstract

The early postnatal period plays a crucial role in the establishment and maturation of the gut microbiome in avian hosts, significantly influencing their metabolic processes and overall health. This study was carried out to characterize the ontogenetic development of fecal microbiota in emus (*Dromaius novaehollandiae*) during critical early growth stages from days 7 to 28 post-hatch using 16S rRNA gene sequencing. The results indicated that rank abundance and rarefaction curves confirmed adequate sequencing depth for capturing microbial diversity across all age groups. The dominant phyla of fecal microbiota in emus included *Firmicutes*, *Proteobacteria*, and *Bacteroidetes*, with successional shifts observed at order, family, and genus levels. As emus advanced in age, fecal microbiota underwent significant changes in microbial community, diversity, and function. The *α*-diversity indices (Observed species, Shannon, PD whole tree, Chao1, and ACE) in the feces of emus peaked significantly at d 21 (*p* < 0.05). The *β*-diversity analysis revealed significant structural differences in microbial communities between different ages, particularly between d14 and d7, d21 vs. d7, and d14 vs. d28 (*p* < 0.05). Linear discriminant analysis Effect Size (LEfSe) identified 26 discriminative biomarkers with stage-specific enrichments, including *Turicibacter* (d7/d28), *Erysipelotrichaceae* (d7/d14), *Bacteroidetes* (d21), and *Corynebacteriaceae* as well as *Actinobacteria* (d28). T-test validation confirmed significant temporal variations in phylum (*Firmicutes*, *Actinobacteria*, and *Bacteroidetes*) and genus-level abundances (e.g., *Bacteroides* and *Lactobacillus*) in the feces of emus (*p* < 0.05). PICRUSt functional prediction indicated age-dependent metabolic pathway enrichment, including amino and nucleotide sugar metabolism (d7), oxidative phosphorylation (d14), ABC transporters and cysteine metabolism (d21), and genetic information processing pathways (d28). These results demonstrated dynamic, stage-specific restructuring of the fecal microbiota and its metabolic potential during early development in emus. This research presented the initial longitudinal assessment of fecal microbiota development in emus throughout their crucial early developmental stage, revealing age-dependent alterations in microbial composition and metabolic activity that could guide enhanced nutritional and health approaches for ratites.

## 1 Introduction

The gut microbiota encompasses a diverse array of microorganisms, such as bacteria, archaea, eukaryotic microbes, and viruses (Combrink et al., 2023). In domestic animals, the dynamic community of gut microbiome plays pivotal roles in maintaining animal growth and health by influencing the host’s nutrient metabolism, gut health, immune modulation, and pathogen resistance in avian species (Pan and Yu, 2014; Aruwa et al., 2021; Gilroy, 2021; Marková et al., 2024). The microbial colonization begins at hatching and undergoes dynamic succession during early development when birds are susceptible to various stressors and diseases (Hines et al., 1995; Madlala et al., 2021), thus affecting their health and survival outcomes (Ruuskanen, 2024). Understanding the dynamics and regulatory mechanisms governing intestinal microbiota in animals can enhance insights into their health and survival, thereby providing valuable guidance for conservation and breeding efforts (Sun et al., 2022). With the development of next-generation sequencing (NGS) technology, the amplicon sequencing targeting the 16S ribosomal RNA (rRNA) gene remains the predominant approach for investigating microbial communities in poultry (Burke and Darling, 2016; Edwards et al., 2023; Lyte et al., 2025). Despite increasing research advances have unveiled host-specific microbial signatures in domesticated poultry species (Fathima et al., 2022; He et al., 2023; Burrows et al., 2025; Lecoeur et al., 2024) and long-lived birds such as ostriches (Videvall et al., 2020), how fecal microbiota assembly correlates with age-specific physiological demands in young emu remains largely unknown.

The emu (*Dromaius novaehollandiae*), is the largest flightless bird native to Australia and is currently farmed globally due to their economic significance with the production of oil, meat, eggs, and leather (Beckerbauer et al., 2001; Sales, 2007; Lindsay et al., 2010; Deshmukh et al., 2021; Koshiishi et al., 2022; Shell et al., 2016). Emu cultivation has recently gained economic prominence with notable expansion increasingly documented across emerging economies in Asian countries such as China and India (Gujjar et al., 2017). Previous study has characterized the growth process of emus as a valuable strategy to optimize feeding and management regimes in the emu industry (Goonewardene et al., 2003). To enhance efficiency, the identification procedures were optimized to accelerate processing while preserving diagnostic precision (Koshiishi and Wada, 2018). Thus, emu offers an ideal model to investigate age-dependent microbial succession. Given their altricial development, extended longevity, and significant growth needs after hatching, microbiome assembly and composition likely dictate physiological outcomes throughout a prolonged developmental window. Prior study has shown the phylogenetic microbial diversity and metabolic function in different segments of the small intestine (duodenum, jejunum, and ileum) (Kim et al., 2023) as well as in the cecum using four adult emus (Bennett et al., 2013; Kim et al., 2023). Since emus usually mature at approximately 1.5 year of age and have a lifespan of 5 to 10 years (Koshiishi et al., 2022), they experience rapid dietary transitions and environmental exposures as precocial hatchlings. However, to the best of our knowledge, there is very little longitudinal study quantifying fecal microbiota dynamics in the first month during their early growth, when their microbial community might be unstable and highly variable.

Feces represent an ideal sampling source for describing the gut microbiota of animals when destructive sampling is not a primary option (Edwards et al., 2023), and has the great advantage for collecting a series of samples from a specific bird over time (Stanley et al., 2015). Hence, this study was carried out to investigate how fecal microbiota changed with age in neonatal emus at early developmental stages. Here, we characterized the taxonomic resolution of dominant microbial consortia in the feces of emu chicks from days 7 to 28 post-hatch using 16S rRNA gene sequencing, aiming to provide a framework to optimize management protocols for threatened ratites worldwide.

## 2 Materials and methods

### 2.1 Experimental design and management

The experimental protocol of the animal study was reviewed and approved by the Animal Care and Use Committee of Foshan University (FOSU2022004). All procedures were performed in accordance with relevant guidelines and regulations.

A total of 8 one-day-old healthy emus (*Dromaius novaehollandiae*) were kindly provided from a commercial farm (Xinhua EMU Cultural Ecological Park, Qingyuan, China). Disinfection of animal housing pens was performed using potassium permanganate and formalin 1 week prior to introducing emu chicks. The emus were kept in an environmental-controlled house with constant temperature of 28–30 °C and relative humidity at 60–70%. Heat lamps were suspended 50–60 cm above the floor on the first day, and then gradually increasing the height according to the ages. The emus were fed with the common complete diets (Table 1) and fresh Napier grass roughage (cut to 1–2 cm lengths) at 1:1 ratio. All birds had free access to diets and water during the whole experiment. The fresh feces were collected separately from each individual emu at the morning of 7, 14, 21 and 28 days of age (*n* = 8), and immediately frozen in liquid nitrogen followed by storage at −80 °C until 16S rRNA gene sequencing analysis. Based on availability and feasibility for a longitudinal study, fecal samples at simple size served as a non-invasive proxy to assess the gut microbiota community dynamics in current study.

**Table 1:** Composition and nutrient levels of the basal diet (air-dry basis).

Item	%
Ingredient	
Corn	54.59
Soybean meal	31.25
Fish meal	3.00
Wheat bran	7.04
Dicalcium phosphate	1.37
Met	0.12
Limestone	1.35
Salt	0.28
Mineral-vitamin premix^1^	1
Nutrient level	
ME, kcal/kg	2,710
Crude protein, %	20.96
Crude fiber, %	3.70
Calcium, %	1.05
Total phosphorus, %	0.77
Available phosphorus, %	0.48
Met, %	0.47
Lys, %	1.17

^1^The premix provided per kg diet: VA 12,000 IU, VD3 4,000 IU, VE, 35 IU, VK3, 4.7 mg, VB1, 1 mg, VB2, 3 mg, VB6, 2 mg, VB12, 0.01 mg, Niacin, 20 mg, calcium pantothenate, 4 mg, folic acid, 1 mg, biotin, 0.05 mg, Fe, 100 mg, Cu, 20 mg, Mn, 100 mg, Zn, 80 mg, I, 3 mg, Se, 0.2 mg.

### 2.2 16S rRNA gene sequencing and data processing

Microbial DNA was isolated from the fecal samples of emus. The V3-V4 hypervariable region of bacterial 16S rRNA genes was amplified employing specific primers (341F: 5’-CCTAYGGGRBGCASCAG-3′; 806R: 5’-GGACTACNNGGGTATCTAAT-3′). Constructed libraries underwent quality assessment and paired-end sequencing (PE250) on an Illumina HiSeq 2,500 system (Novogene Bioinformatics Technology Co., Ltd., Tianjin, China). Subsequent bioinformatic processing utilized the QIIME software package (v1.9.1) for quantitative microbial ecology assessment. Operational taxonomic units (OTUs) were delineated by clustering sequences at a 97% similarity threshold. Relative abundances for the Top 10 bacterial taxa across phylum, class, order, and genus classifications were derived using the taxa plugin. The *α*-diversity was quantified via metrics including Observed_species, Shannon, Simpson, Chao1, ACE, and PD_whole_tree indices. Treatment effects on microbial community structure (*β*-diversity) were evaluated using non-metric multidimensional scaling (NMDS), UPGMA clustering, analysis of similarities (ANOSIM), and β-diversity index. Differential abundance of microbiota between treatments was statistically compared using the linear discriminant analysis effect size (LEfSe) method and T-tests. The datasets presented in this study can be found in the NCBI Bioproject repository^1^ under project accession number PRJNA1331932.

### 2.3 Statistical analyses

The data were analyzed using one-way ANOVA followed by Duncan’s multiple comparison test for post-hoc analysis in SPSS software (version 26.0; IBM Corp., Armonk, NY). Data are presented as means ± standard error (SE). Differences were considered statistically significant at *p* < 0.05.

## 3 Results

**Figure 1:**
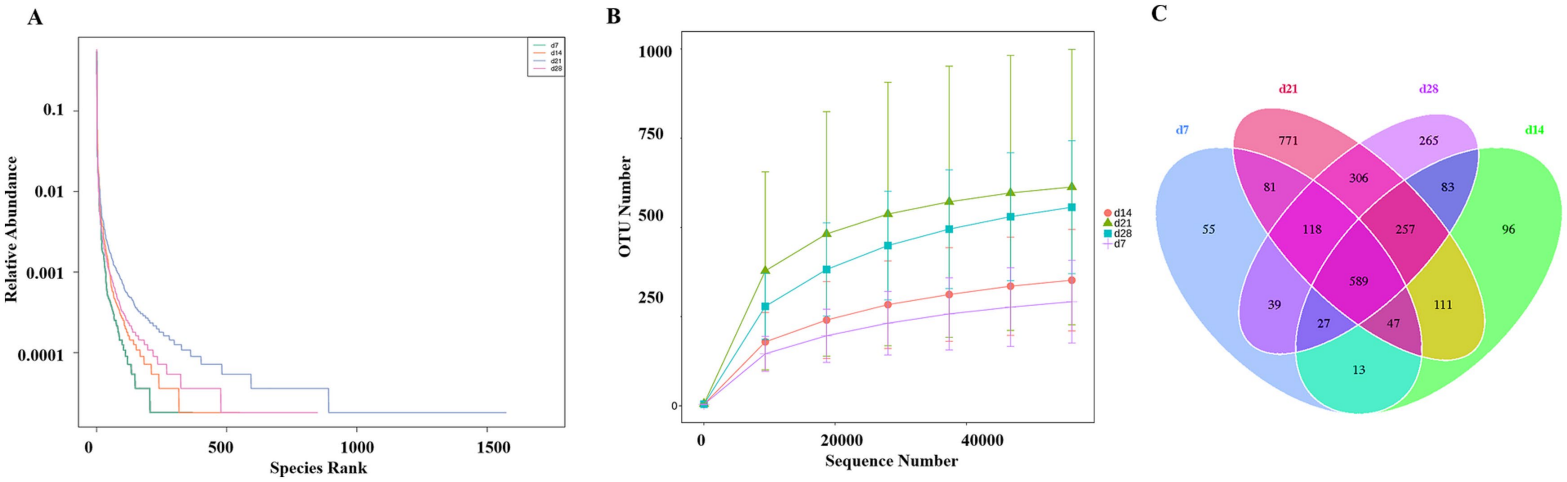
The rank abundance, rarefaction curve, and Venn plot of fecal microbiota in emus with different ages: **(A)** Rank abundance; **(B)** Rarefaction curve; **(C)** Venn plot.

The Rank abundance analysis (Figure 1A) and Rarefaction curve analysis (Figure 1B) demonstrated that sequencing depth was adequate, with curves approaching saturation, confirming comprehensive coverage of bacterial diversity across all experimental groups. The Venn diagram (Figure 1C) further identified 589 operational taxonomic units common to all four treatments in the emu feces, with 55, 96, 771, and 265 unique OTUs at d 7, 14, 21, and 28, respectively. These findings collectively indicate sufficient sequencing depth captured the predominant microbial diversity present in the fecal samples of emus.

**Figure 2:**
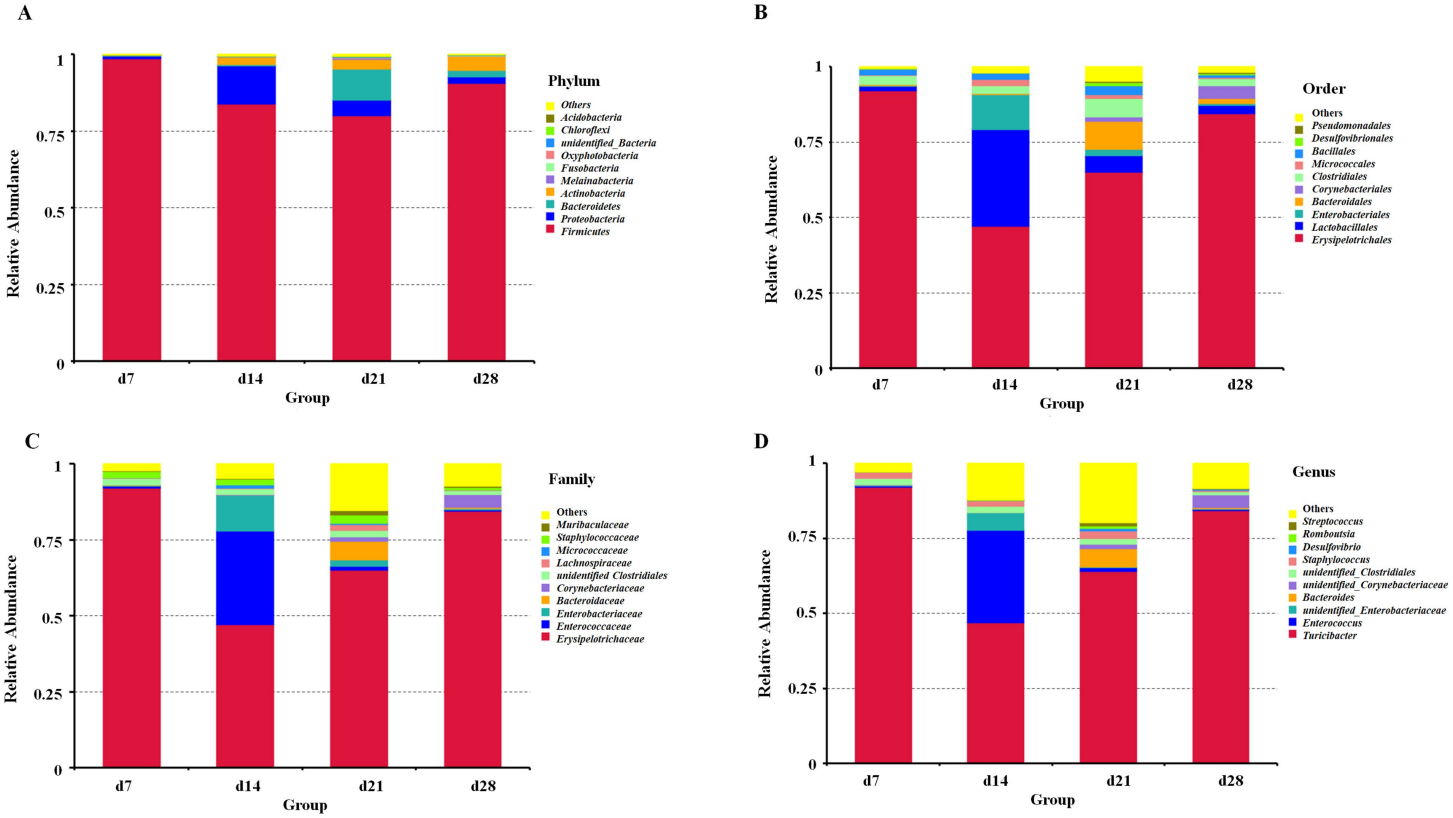
The microbial community structure in the feces of emus with different ages: **(A)** phylum, **(B)** order, **(C)** family, **(D)** genus.

The top 10 predominant bacteria abundances at the phylum, order, family, and genus are shown in Figure 2. Specially, the top 10 dominant phyla included *Firmicutes*, *Proteobacteria*, *Bacteroidetes*, *Actinobacteria*, *Melainabacteria*, *Fusobacteria*, *Oxyphotobacteria*, *unidentified_Bacteria*, *Chloroflexi*, and *Acidobacteria* (Figure 2A). At the order level, the top 10 dominant orders were *Erysipelotrichales*, *Lactobacillales*, *Enterobacteriales*, *Bacteroidales*, *Corynebacteriales*, *Clostridiales*, *Micrococcales*, *Bacillales*, *Desulfovibrionales*, and *Pseudomonadales* (Figure 2B). Furthermore. At the family level, *Erysipelotrichaceae*, *Enterococcaceae*, *Enterobacteriaceae*, *Bacteroidaceae*, *Corynebacteriaceae*, *unidentified Clostridiales*, *Lachnospiraceae*, *Micrococcaceae*, *Staphylococcaceae*, and *Muribaculaceae* were identified as the top 10 families (Figure 2C). Moreover, the top 10 dominant genera included *Turicibacter*, *Enterococcus*, *unidentified_Enterobacteriaceae*, *Bacteroides*, *unidentified_Corynebacteriaceae*, *unidentified_Clostridiales*, *Staphylococcus*, *Desulfovibrio*, *Romboutsia*, and *Streptococcus* (Figure 2D).

**Figure 3:**
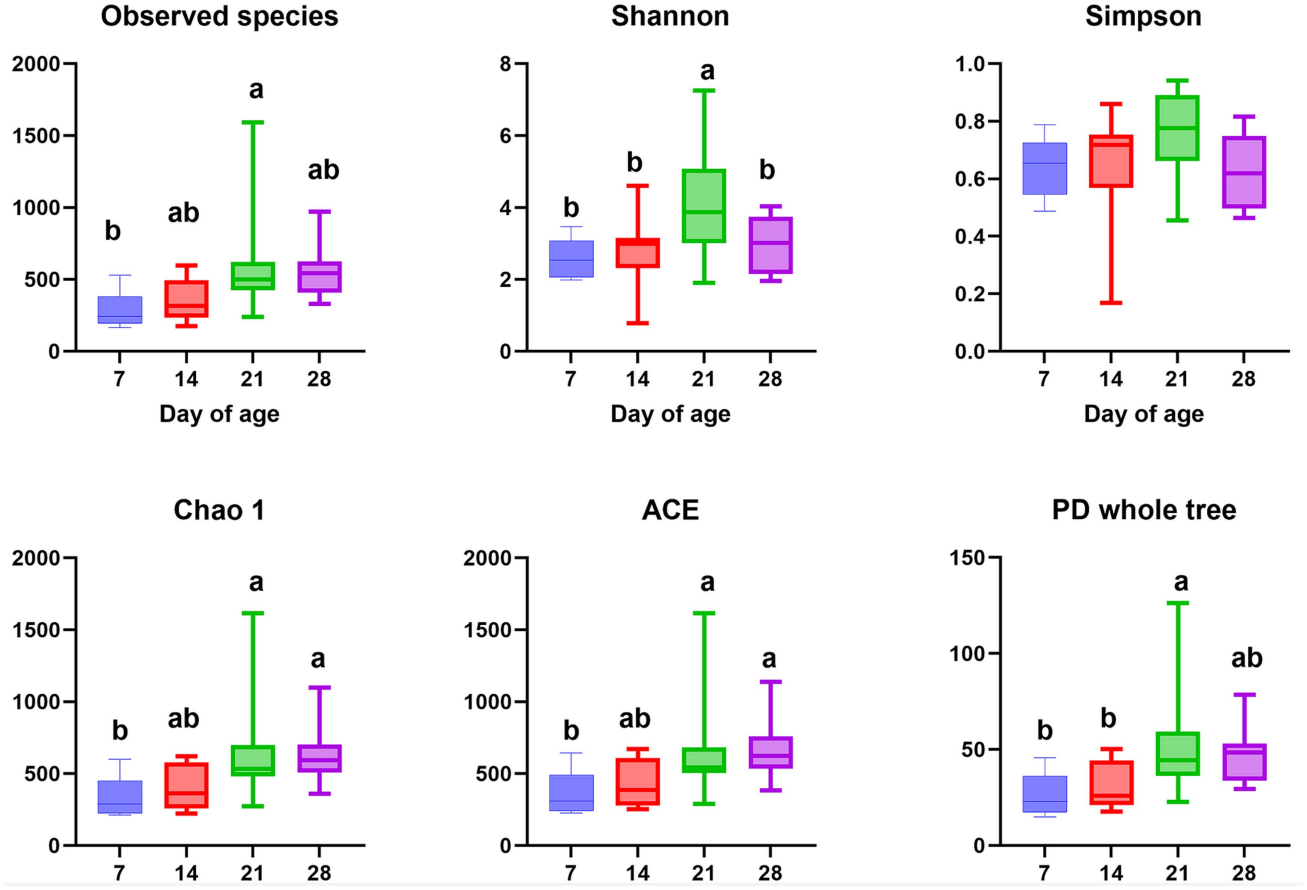
The microbial *α*-diversity in the feces of emus with different ages (a, b). Means in the columns with different superscripts differ (*p* < 0.05).

**Figure 4:**
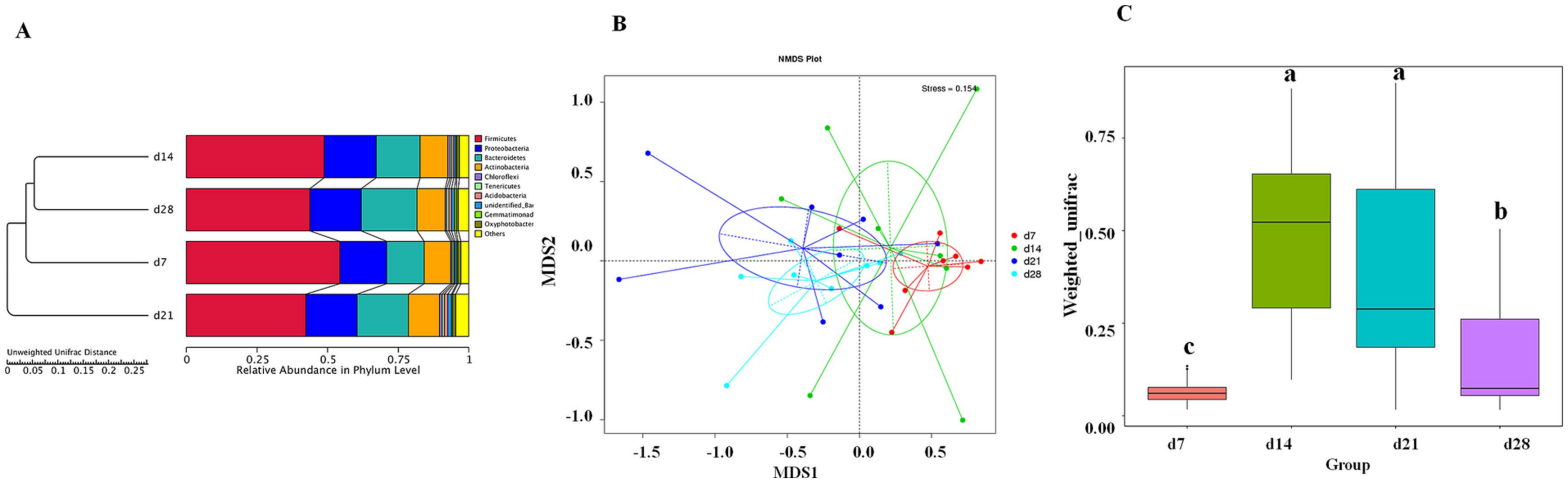
The microbial *β*-diversity of fecal microbiota in emus with different ages. **(A)** UPGMA analysis based on unweighted unifrac distance. **(B)** NMDS plot. **(C)** β-diversity index of fecal microbiota based on weighted unfirac distance using Wilcox method.

**Figure 5:**
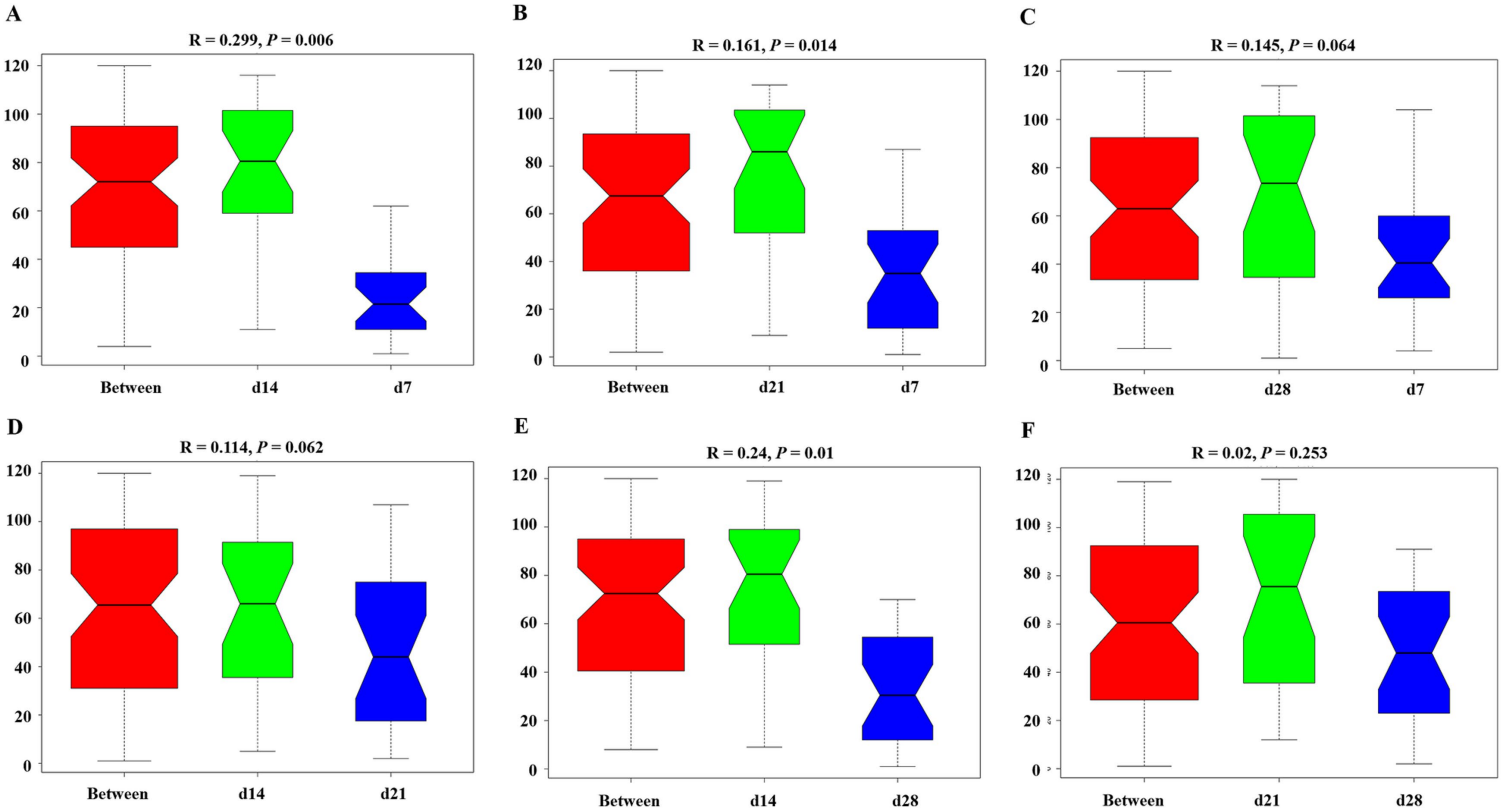
The analysis of similarities (ANOSIM) tests of fecal microbiota in emus with different ages. The ANOSIM tests were performed between groups based on relative abundance of OUT. **(A)** d 14 vs. d 7; **(B)** d 21 vs. d 7; **(C)** d 28 vs. d 7; **(D)** d 14 vs. d 21; **(E)** d 14 vs. d 28; **(F)** d 21 vs. d28.

The *α*-diversity of gut microbiota in the feces of emu at different days of age are shown in Figure 3. The Observed_species, Shannon, and PD whole tree indices of emu feces at d 21 were higher than those in the other groups (*p* < 0.05). Moreover, the Chao 1 and ACE indices of emu feces were significantly increased at d 21 and 28 when compared to d 7 (*p* < 0.05). The UPGMA (Figure 4A) and NMDS (Figure 4B) analyses revealed distinct differences of microbial community among different days of age in emu feces. Moreover, the *β*-diversity index of the emu fecal microbiota at d 14 and 21 were significantly higher than d 7 and 28 (*p* < 0.05; Figure 4C). Additionally, the ANOSIM analysis (Figure 5) showed that the microbial community structures of the emu feces were significantly different between d 14 group and d 7 group (*R* = 0.299, *p* = 0.006), and between d 21 group and d 7 group (*R* = 0.161, *p* = 0.014), as well as between d 14 group and 28 group (*R* = 0.24, *p* = 0.01) in the feces of emu.

**Figure 6:**
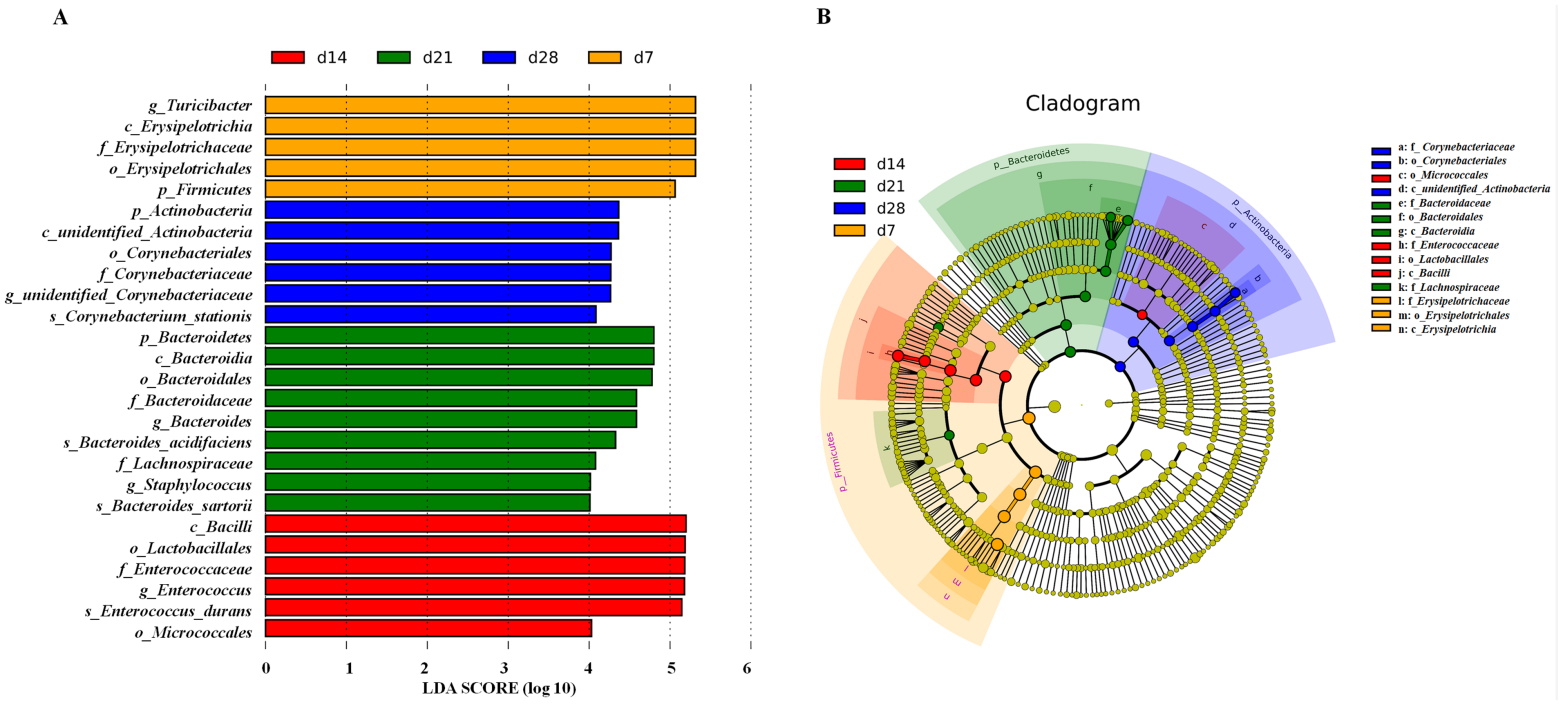
Linear discriminant analysis (LDA) effect size (LEfSe) of fecal microbiota in emus with different ages. **(A)** LEfSe analysis (LDA ≥ 4). **(B)** LEfSe cladogram.

**Figure 7:**
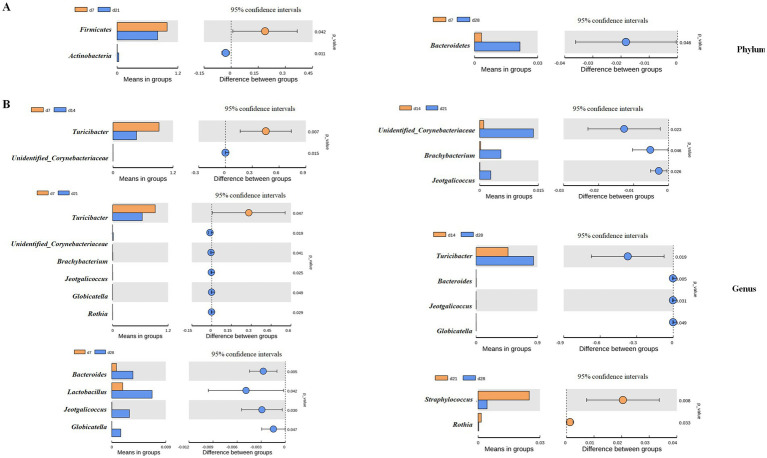
*T*-test analysis for the differential bacterial of fecal microbiota in emus with different ages: **(A)** Phylum and **(B)** Genus.

The differences in taxonomic abundances between treatments were compared by further LEfSe (Figure 6) and T-test analyses (Figure 7). There were 26 discriminative biomarkers identified among different days of age in the feces of emu (Figure 6A). Specially, 5 bacterial taxa including g_*Turicibacter*, c_*Erysipelotrichia*, f_*Erysipelotrichaceae*, o_*Erysipelotrichales*, and p_*Firmicutes* were significantly enriched at d 7 of age in the feces of emus. Furthermore, p_*Actinobacteria*, *c_unidentified_Actinobacteria*, *o_Corynebacteriales*, *f_Corynebacteriaceae*, *g_unidentified_Corynebacteriaceae*, and *s_Corynebacterium_stationis* were abundant at d 28 of age in the feces of emu. There were 10 bacterial taxa including p_*Bacteroidetes*, c_*Bacteroidia*, o_*Bacteroidales*, f_*Bacteroidaceae*, g_*Bacteroides*, s_*Bacteroides_acidifaciens*, f_*Lachnospiraceae*, g_*Staphylococcus*, and s_*Bacteroides_sartorii* enriched at d 21 of age in the feces of emus. Moreover, c_*Bacilli*, o_*Lactobacillales*, f_*Enterococcaceae*, g_*Enterococcus*, s_*Enterococcus_durans*, and o_*Micrococcales* were abundant at d 14 of age in the feces of emus. The cladogram of LEfSe enriched the microbial biomarkers mainly belonging to the three phyla, including *Firmicutes*, *Bacteroidetes*, and *Actinobacteria* (Figure 6B).

Moreover, the bacteria differentially enriched by T-test are shown in Figure 7. At the phylum level, the relative abundance of *Firmicutes* at d 21 was decreased while that of *Actinobacteria* was increased at d 21 when compared to d 7 in the feces of emu (*P*<0.05). In addition, the relative abundance of Bacteroidetes at d 7 was decreased when compared to d 28 in the feces of emu (*p* < 0.05). At the genus level, the relative abundance of Turicibacter in feces of emu was significantly increased at d 7 and 28 when compared to d 14 and 21, while the relative abundances of *Bacteroides*, *Lactobacillus*, *Jeotgalicoccus*, and *Globicatella* were increased at d 28 in relative to d 7 or d 14 (*p*<0.05). Moreover, in contrast to d 14 group, the relative abundances of *Unidentified_Corynebacteriaceae*, *Brachybacterium*, and *Jeotgalicoccus* were all increased at d 21 in the feces of emu (*p* < 0.05). These results collectively suggests that the microbial communities that play important roles in different growth stages of emu are different.

**Figure 8:**
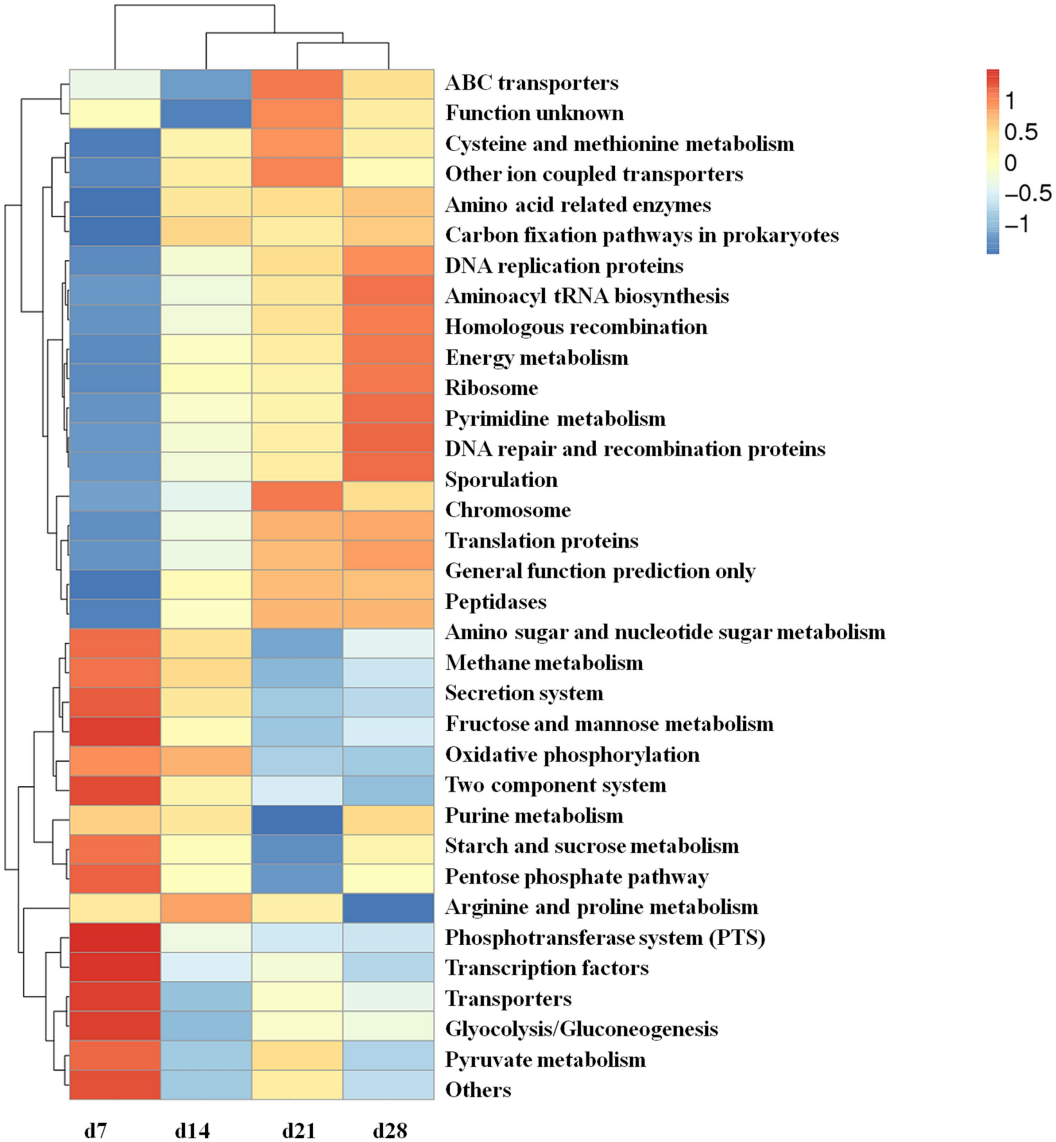
Predicted functional profile at the third level of the microbiome in feces of emus with different ages.

The PICRUST functional prediction analysis was performed on the fecal microbiota of emu at d 7, 14, 21, and 28 of age to better understand the role of microbial communities in the host. There were significant differences in the metabolic pathways of KEGG Level 3 among the emus of different ages (Figure 8). The results showed that compared with the other groups, the fecal microbes of 7-day-old emu had high levels of metabolic pathways including amino sugar and nucleotide sugar metabolism, methane metabolism, secretion system, fructose and mannose metabolism, oxidative phosphorylation, two component system, starch and sucrose metabolism, pentose phosphate pathway, phosphotransferase system (PTS), transcription factors, transporters, glyocolysis or gluconeogenesis, and pyruvate metabolism. Moreover, oxidative phosphorylation as well as arginine and proline metabolism pathways were enriched at 14-day-old emu compared to d 21 and 28. For the 21-day-old emu, the pathways of ABC transporters, cysteine and methionine metabolism, other ion coupled transporters, and chromosome were significantly increased when compared to 7-d-old emu. Furthermore, the DNA replication proteins, aminoacyl tRNA biosynthesis, homologous recombination, energy metabolism, ribosome, pyrimidine metabolism, DNA repair and recombination proteins, and sporulation when compared to other groups.

## 4 Discussion

The gut microbiota is a complex ecological community comprising of commensal, symbiotic, and pathogenic microorganisms, which is closely associated with performance, productivity, and condition in poultry (Marková et al., 2024). Microbial communities have been well recognized to play significant roles in impacting nutrition, immune function, and physiological responses in avian hosts (Kohl, 2012). The development of gut microbiota in animals is collectively governed by age (growth stages), diets, genetic background, management, environmental exposures, and antibiotics, which critically determine microbial community structure, metabolic function, and host health outcomes (Sun et al., 2022; Tang et al., 2025). Indeed, the characteristics of intestinal microbial communities, such as their composition, diversity, and functionality, remain dynamic and undergo continuous changes over time (Anwar et al., 2021). For example, previous studies have clarified the dynamic changes in the gut microbial community and function during embryonic development of chicken (Akinyemi et al., 2020) and later broiler growth (Yang et al., 2022). Furthermore, previous studies have documented the age-related dynamics in the gut microbiome of pigs (Wang et al., 2019), cows (Guo et al., 2020), ducks (Zhu et al., 2020), and ostriches (Li et al., 2020). A stable gut microbiota enhances host resistance to colonization by pathogenic microorganisms, thereby reducing susceptibility to infections (Fuller, 1989; Umetsu et al., 2002). The initial developmental phase represents a vital period for the establishment and progression of microbial communities, wherein disruptions may lead to significant and enduring consequences on host health. However, the longitudinal investigation data on the fecal microbial community and diversity of emus (*Dromaius novaehollandiae*) particularly during early developmental stages remain limited. This research further examines the developmental changes of fecal microbial community in the first month of emus, with the goal of supporting intestinal equilibrium and improving resistance to illness, ultimately lowering fatality rates in emus.

This study conducted 16S rRNA gene sequencing analysis on the feces of emus at early life, and found that *Firmicutes*, *Proteobacteria*, *Bacteroidetes*, and *Actinobacteria* were the dominant phyla microorganisms in the feces of emus at different weeks during the first month. This was in consistent with prior study reporting *Firmicutes* (14–99%) and *Proteobacteria* (0.5–76%) as the most predominant bacterial phyla in the small intestine of emus (Kim et al., 2023). However, another study has shown that the most predominant bacterial phyla were *Bacteroidetes* (56.8%), *Proteobacteria* (23.6%), *Fusobacteria* (11.29%), and *Firmicutes* (7.0%) in the cecum of adult emus (Bennett et al., 2013). Notably, previously reported studies of gut microbiota results in emus were from comparatively small sample sizes (Bennett et al., 2013; Kim et al., 2023). Moreover, fecal microbiota was qualitatively similarly to cecal microbiota but quantitatively different in poultry (Stanley et al., 2015). Here, we found that fecal microbiota in young emus showed a marked reduction in *Firmicutes* and a concurrent rise in *Actinobacteria* by d 21 relative to d 7, while *Bacteroidetes* abundance was significantly lower at d 7 than d 28. Importantly, *Firmicutes* play vital role in fermentative metabolism of plant polysaccharides that could help the emus digesting dietary fiber to support the maintenance energy requirement of emus (Herd and Dawson, 1984). Moreover, *Bacteroidetes* are also known to specialize in breaking down complex plant polysaccharides (Flint et al., 2008), while Actinomycetota are distinguished by their broad capacity for secondary metabolite synthesis (Barka et al., 2016). As emus develop, shifts in nutritional requirements appear to drive microbial community restructuring, potentially enhancing metabolic and digestive functions suited to each growth phase. Indeed, gut microbiota undergoes dynamic shifts during different growth phases, and ages represents an important factor affecting the cecal microbiota as reported in Wenchang chickens (He et al., 2023). A similar study in broilers indicated that gut microbiota displayed unique compositional signatures at distinct life stages (Li et al., 2022). Previous study has also shown that *Bacteroidetes*, *Firmicutes*, *Actinobacteria*, *Proteobacteria*, and *Spirochaetes* were the predominant phyla at d 21 of age in chicken, with the abundances of *Firmicutes* and *Actinobacteria* rising with the increasing age of chicks (Thomas et al., 2019). Additionally, the *Proteobacteria* was the dominant phyla in young layers at the first week, which abundance was then decreased to less than 10% from the second to fourth week of life (Videnska et al., 2014). Consistently, the relative abundance of *Firmicutes* were decreased while *Bacteroidetes* and fiber-degrading bacteria were significantly increased in layer chickens from 8 to 50 weeks of age (Sun et al., 2021). The results from our and other investigators indicated that the gastrointestinal tract harbors a diverse community in emus, and dynamic developmental restructuring of the core microbiome changed over age.

Additionally, the present study found that at the genus level, *Turicibacter*, *Enterococcus*, and *Bacteroides* were dominant in emu feces, and shifted significantly with ages. Specially, the current observed temporal succession, characterized by the progressive colonization of taxa such as *Enterococcus* (d14), *Bacteroides* (d 21), and *Corynebacteriaceae* (d 28), suggested that each microbial assemblage might perform specialized functions aligned with the changing physiological demands during emu development. Interestingly, *Turicibacter* has been shown to regulate the host bile acid and lipid metabolism that could affect body weight changes (Lynch et al., 2023). Furthermore, *Enterococcus* are typical commensal intestinal bacteria, but can also act as opportunistic pathogens contributing to the occurrence of enterococci in poultry (Souillard et al., 2022). Moreover, *Bacteroides* exhibited beneficial effects by helping degrading the indigestible carbohydrates (Salyers et al., 1977). Notably, higher levels of *Bacteroides* in the early life of chickens might be advantageous, particularly for boosting short-chain fatty acid output and promoting intestinal health with reduced inflammation (Fan et al., 2023). Evidence suggests the role of *Bacteroidetes* is divergent and age-specific, exhibiting a favorable relationship in younger populations that becomes adverse in older adults (Marková et al., 2024). Previous study in ostriches also demonstrated that the abundance of the genus *Bacteroides* in the feces was positively correlated with the growth of ostriches during the first week of age (Videvall et al., 2019). Furthermore, families including *Peptococcaceae*, *S24-7*, *Verrucomicrobiae*, *Anaeroplasmataceae*, *Streptococcaceae*, *Methanobacteriaceae* abundances at the first 2 weeks associated with subsequent reductions in growth of ostriches in an age-specific and transient manner (Videvall et al., 2019). This could be explained by the close evolutionary kinship and their analogous trajectories of microbial colonization between the ostriches and emus.

Besides the microbial community composition, the present study also found that the fecal microbial*α*-diversity and *β*-diversity of emu at different ages significantly differed. Current α-diversity analysis indicated that the Observed species, Shannon, Simpson, Chao 1, ACE, and PD whole tree indices of fecal microbiota in emus increased from d 7 to d21, followed by a slight decline by d 28. Notably, d 21 appears to represent a key ecological transition point within the fecal microbial community of emus, accompanied with a notable increase in *Bacteroidetes* abundance mentioned above. The observed pattern, in which α-diversity stabilized or experienced a slight decline after an initial peak, aligned with trends reported in other species. For example, a previous study in broilers showed that the α-diversity of the gut microbiota exhibited a gradual reduction between weeks 4 and 16 (Yang et al., 2022). Another study in ducks have also documented dynamic changes in microbial α-diversity and β-diversity, indicating significant shifts in species richness and community structure along the intestinal tract and throughout development, accompanied by temporal variations in bacterial composition (Zhu et al., 2020). Moreover, significant disparities in β-diversity index among different ages of emus indicated that each developmental period harbored a specific microbial ecosystem during the first month after hatch. Interestingly, the Chao 1 index in the cecum has been reported to be higher than that in the small intestines including duodenum, ileum, and jejunum of the emus (Kim et al., 2023). This might be attributed to the fact that the cecum of emu provides a more stable environment characterized by longer digesta retention times and nutrient richness, which favored the colonization and proliferation of diverse microbial communities when compared to small intestine (Kim et al., 2023). Furthermore, Videvall et al. (2019) observed a progressive rise in microbial diversity during ostrich chick development, marked by repeated colonization and extinction dynamics as well as a significant taxonomic reorganization of bacteria, which aligned temporally with the completion of yolk absorption. Indeed, evidence has shown close association between host growth rate and the diversity of the gut microbiota (Zhu et al., 2021). Additional investigations showed that behavior of coprophagy in ostrich juveniles increased gut microbial diversity that strongly correlated with enhanced host health and improved defense mechanisms against pathogenic invasions (Videvall et al., 2023). Interestingly, emus are prone to coprophagia that could contribute to the presence of protozoan parasites in the gastrointestinal tract and feces (Gallo et al., 2020). This present finding aligned with earlier reports, indicating that the fecal microbiota of emus undergoes progressive changes correlated with age, resulting in substantial reorganization of microbial composition and diversity.

Furthermore, the functional profiling via PICRUSt analysis revealed stage-specific enrichment of metabolic pathways in emu fecal microbiota, especially enriching the pathways of amino and nucleotide sugar metabolism at d 7, oxidative phosphorylation at d 14, ABC transporters and cysteine metabolism at d 21, and genetic information processing pathways at d 28. These stage-specific biomarkers and metabolic signatures provide novel tools for health assessment and inform timely microbial interventions in avian conservation programs. Consistently, other researchers used PICRUSt-based functional predictions showed detoxification pathways enriched in the small intestine microbiota of emus, while the cecal microbiome displayed enhanced protective and immune responses against enteric pathogens (Kim et al., 2023). This indicates that functional microbial communities involved in fiber digestion remain active even under anatomically constrained fermentation conditions. Plant fiber degradation is shaped by gut microbiota composition, retention time in fermentative compartments, and cell wall structure (Flint et al., 2012). The relatively shorter cecum of young emus likely limits their capacity for prolonged plant material retention, creating a less favorable environment for microbes reliant on extended fermentation (Herd and Dawson, 1984). This anatomical constraint, however, appears evolutionarily counterbalanced by efficient foregut digestion and prolonged chyme retention in the ileum and rectum (Herd and Dawson, 1984; Smith et al., 2009). Gastric pepsin can hydrolyze hemicellulose under acidic conditions (Parra, 1978), supported by reduced metabolic demands (Calder and Dawson, 1978) and highly effective foregut processing. Collectively, the observed age-related shifts in key metabolic pathways underscore the functional plasticity of the gut microbiota, which aligns with critical stages of host development and contributes to emu growth and survival.

Although fecal samples provide only a partial representation of the entire gut microbiota, and the limited sample size in this study warrants caution in generalization, they remain a valuable non-invasive tool for tracking broad microbial community dynamics in emus. Therefore, the successional progression observed in the structure, diversity, and functional capacity of the fecal microbiome in neonatal emus revealed stage-specific dynamics during this critical early-life window, offering meaningful reference data for future investigations.

## 5 Conclusion

This study explored dynamic changes in fecal microbiota composition, diversity, and microbial metabolic function of emus during the colonization process of early growth stages from 7 to 28 days of age. These adaptations may align with host physiological transitions during development to support the well-being of emus. To our knowledge, this is the first longitudinal study to characterize the fecal microbiome development in emus during this critical window, providing important guidance for refining nutritional management and future research concerning microbial ecology in ratites. Further studies with larger sample sizes should be carried out to elucidate the potential interactions between the microbiome and growth and development, immune function, and gut health of emus.

## Data Availability

The data presented in this study are publicly available. This data can be found at: https://www.ncbi.nlm.nih.gov, accession PRJNA1331932.

## Footnotes

1
http://www.ncbi.nlm.nih.gov/bioproject
